# Ethical and legal considerations in healthcare AI: innovation and policy for safe and fair use

**DOI:** 10.1098/rsos.241873

**Published:** 2025-05-14

**Authors:** Tuan Pham

**Affiliations:** ^1^ Barts and The London School of Medicine and Dentistry, London, UK

**Keywords:** artificial intelligence, healthcare, ethics, laws and regulations, policies

## Abstract

Artificial intelligence (AI) is transforming healthcare by enhancing diagnostics, personalizing medicine and improving surgical precision. However, its integration into healthcare systems raises significant ethical and legal challenges. This review explores key ethical principles—autonomy, beneficence, non-maleficence, justice, transparency and accountability—highlighting their relevance in AI-driven decision-making. Legal challenges, including data privacy and security, liability for AI errors, regulatory approval processes, intellectual property and cross-border regulations, are also addressed. As AI systems become increasingly autonomous, questions of responsibility and fairness must be carefully considered, particularly with the potential for biased algorithms to amplify healthcare disparities. This paper underscores the importance of multi-disciplinary collaboration between technologists, healthcare providers, legal experts and policymakers to create adaptive, globally harmonized frameworks. Public engagement is emphasized as essential for fostering trust and ensuring ethical AI adoption. With AI technologies advancing rapidly, a flexible regulatory environment that evolves with innovation is critical. Aligning AI innovation with ethical and legal imperatives will lead to a safer, more equitable healthcare system for all.

## 1. Introduction

Artificial intelligence (AI) is set to revolutionize healthcare, transforming the landscape of medical practice and patient care in ways that were once unimaginable [1,2]. The potential of AI to enhance diagnostic accuracy, optimize treatment plans, streamline healthcare operations and improve patient outcomes has attracted significant attention from medical professionals, researchers and policymakers alike. With its ability to analyse vast amounts of data, detect hidden patterns and provide real-time insights, AI has the power to redefine how healthcare is delivered across the globe.

One of the most profound applications of AI in healthcare is in diagnostics [3–5], where machine learning algorithms are being deployed to interpret medical data, such as medical imaging, lab results, and patient histories, more efficiently and accurately than traditional methods. The ability of AI to process medical images—such as X-rays, magnetic resonance imaging (MRI) and computed tomography (CT) scans—has already shown promise in detecting diseases like cancer, cardiovascular conditions and neurological disorders, often identifying them earlier than human clinicians can. AI-powered diagnostic systems can analyse patterns in large datasets that would otherwise go unnoticed, enabling healthcare providers to catch diseases at their earliest stages when treatment is most effective [6].

The field of personalized medicine stands to benefit significantly from AI advancements. By integrating data from various sources—genetic profiles, lifestyle habits, environmental factors and clinical history—AI systems can provide individualized treatment plans tailored to each patient’s specific needs [7–9]. This shift towards precision medicine has the potential to optimize therapeutic efficacy and minimize the side effects of treatments. For instance, AI can predict how a patient might respond to a particular medication based on their genetic markers, ensuring that the right treatment is administered to the right patient at the right time [10]. This approach not only improves outcomes but also leads to more efficient healthcare delivery by reducing unnecessary treatments and hospitalizations.

AI is also revolutionizing robotic surgery [11–14], where advanced algorithms and machine learning are providing surgeons with tools that improve precision, minimize human error and enhance the overall surgical experience. AI-powered robots can assist in performing minimally invasive procedures with unparalleled accuracy, reducing the risk of complications, speeding up recovery times and offering patients a more comfortable experience. These systems can also provide surgeons with real-time feedback and predictive analytics, helping to optimize their decisions during procedures and ultimately improving patient safety.

In medical imaging, AI is driving significant progress by enabling faster, more accurate interpretation of diagnostic images [15–18]. AI tools can now detect subtle abnormalities in scans that may go unnoticed by human clinicians, making them an invaluable resource in identifying early signs of disease. This capability is particularly critical in areas such as oncology, where early detection can drastically improve survival rates. Furthermore, the ability of AI to learn from diverse datasets [19] and continuously refine its models [20] means that its diagnostic capabilities are likely to improve over time, becoming even more reliable and precise.

While the benefits of AI in healthcare are clear, its rapid development brings with it a host of challenges, particularly related to ethical and legal considerations [21,22]. As AI systems become more integral to medical decision-making, it is crucial to ensure that these technologies are used in ways that are both ethical and responsible. Patient privacy, for instance, is a significant concern in the deployment of AI, especially given the vast amount of personal and sensitive data these systems require to function effectively. AI systems must be designed to comply with stringent data protection laws [23], such as the General Data Protection Regulation (GDPR) in the European Union (EU; https://gdpr-info.eu/) or the Health Insurance Portability and Accountability Act (HIPAA) in the US (https://www.hhs.gov/hipaa/index.html), to ensure that patient information is kept secure and confidential.

Moreover, as AI models are trained on historical data, there is the potential for algorithmic bias [24,25], where AI systems may inadvertently perpetuate existing disparities in healthcare outcomes. For example, if an AI model is trained on data that disproportionately represents certain demographic groups, it may fail to accurately diagnose or treat patients from under-represented groups. Addressing these biases through more inclusive data collection and algorithm development is a critical challenge that must be tackled to ensure that AI systems promote fairness and equity in healthcare.

The issue of accountability is another pressing concern. As AI becomes more autonomous in healthcare decision-making, it is increasingly difficult to assign responsibility for errors or adverse outcomes [26–28]. In cases where AI algorithms make incorrect diagnoses or suggest harmful treatments, determining who is liable—the healthcare provider, developers of the AI system, or the hospital—can be legally complex. Clear frameworks must be established to define liability and ensure that AI systems are held to the same standards of accountability as human clinicians.

One of the most promising developments in healthcare AI is the rise of autonomous diagnostic systems [29–32]. These AI systems have the potential to analyse medical data (e.g. radiographs, lab results or EHRs) and make diagnostic decisions with minimal human intervention. While these technologies promise to improve the speed and accuracy of diagnoses, they also raise concerns about the extent of their autonomy and the need for clear guidelines on human oversight. Ethical challenges include ensuring that these systems do not replace human judgement and maintaining mechanisms for accountability when errors occur.

As AI continues to evolve, it is clear that robust ethical and legal frameworks are needed to guide its adoption in healthcare settings [2,28,33]. These frameworks must address concerns such as informed consent, data privacy, algorithmic transparency and patient autonomy. They must ensure that AI is used to enhance, rather than replace, the role of healthcare professionals, with human oversight remaining a central principle of patient care. Regulatory bodies and policymakers have a critical role to play in ensuring that these ethical and legal standards are met while encouraging innovation and progress in the field.

The role of policymakers is fundamental to ensuring the safe and effective integration of AI into healthcare systems [34,35]. As AI technologies develop rapidly, policymakers must keep pace by creating regulations that protect patients without suppressing innovation. This includes establishing guidelines for AI system testing, approval and deployment in clinical settings, as well as developing standards for training healthcare professionals to use AI tools responsibly. Policymakers also need to work with interdisciplinary teams—comprising ethicists, healthcare providers, data scientists and legal experts—to provide adaptive policies that are flexible enough to accommodate future AI advancements while maintaining patient safety, equity and trust.

Furthermore, policymakers must foster international collaboration to ensure that AI applications in healthcare are guided by consistent standards across borders [36,37]. This is particularly important in the context of global health disparities, where AI has the potential to either worsen or reduce inequities in access to care. International partnerships can help create global frameworks for AI ethics, privacy laws and regulatory approval processes, ensuring that the benefits of AI are distributed equitably and responsibly across the world.

This work provides a comprehensive and integrative perspective that distinguishes it from many similar reviews on the topic. While many articles address specific ethical or legal aspects of AI in healthcare [38–42], this review uniquely bridges the gap by simultaneously examining ethical principles, legal frameworks and policy implications within a unified narrative.

Actionable frameworks and guidelines are also proposed to address key challenges, including global regulatory harmonization, adaptive regulations and public engagement strategies, emphasizing the critical role of multidisciplinary collaboration. Unlike other reviews that focus on specific geographies or narrow AI applications, this work adopts a global perspective, drawing comparisons across jurisdictions and analysing cross-border regulatory challenges.

Furthermore, insights are synthesized to highlight the long-term implications of increasing AI autonomy in clinical decision-making, an area that remains relatively under-explored in other reviews. This holistic approach ensures the review offers fresh insights and practical recommendations for stakeholders, including policymakers, technologists and healthcare providers.

As a narrative review, this paper focuses on integrating knowledge from diverse sources to provide a comprehensive overview of ethical and legal considerations in healthcare AI. To ensure rigour, widely recognized references and cutting-edge discussions from recent literature are included. The selection process emphasizes relevance, credibility and contributions to the field, enabling the identification of gaps and the proposal of actionable recommendations.

## 2. AI ethics principles: a systematic perspective

AI ethics principles offer valuable insights into the broader ethical framework necessary for understanding the role of AI in healthcare.

Floridi *et al*. [43] and Jobin *et al*. [44] have identified recurring themes including transparency, accountability, fairness and privacy as central to AI ethics across disciplines and regions. Floridi *et al*. [43] emphasized the importance of translating abstract ethical principles into actionable frameworks, particularly for sectors like healthcare where social good is paramount. The authors stressed that ethical AI systems should not only comply with existing guidelines but also anticipate the potential societal impact of their deployment. Jobin *et al*.’s [44] comprehensive analysis of global AI ethics guidelines revealed a remarkable degree of consensus on core ethical principles while highlighting variations in their prioritization depending on cultural and regional contexts, pointing to the need for localized interpretations of global ethical standards.

Specific articles further elaborate on individual principles, offering actionable insights into the ethical challenges posed by AI in healthcare. Transparency and explainability, as discussed by Miller [41], are critical for fostering trust among patients and providers, particularly when AI systems are involved in decision-making processes. Transparency ensures that the rationale behind AI-driven decisions is comprehensible not only to clinicians but also to patients, empowering them to make informed choices. Explainability becomes even more critical in high-stakes scenarios, such as when AI systems provide diagnostic recommendations or treatment pathways that deviate from traditional clinical reasoning.

Fairness and bias mitigation, explored by Obermeyer *et al*. [39], address the risks of perpetuating healthcare disparities through biased algorithms. Their work highlights how biases embedded in training datasets can amplify systemic inequalities, disproportionately affecting marginalized populations. Addressing such disparities requires the implementation of fairness-aware design, rigorous algorithmic audits and the inclusion of diverse and representative datasets.

Accountability, a recurring theme in the work of Gerke *et al*. [40], emphasizes the importance of establishing clear lines of responsibility for decisions made by AI systems, particularly in high-stakes healthcare environments. With AI systems increasingly being integrated into clinical workflows, ensuring accountability requires redefining legal and professional norms. Questions around liability, such as whether errors in AI-assisted diagnoses are attributable to clinicians, developers or the institutions deploying the technology, highlight the complexity of accountability in hybrid human–AI decision-making processes. Without clear accountability frameworks, the deployment of AI in critical healthcare functions risks undermining trust and ethical integrity.

These foundational works underscore the need for a comprehensive approach that combines ethical principles with practical considerations to address the specific challenges posed by healthcare AI. This study acknowledges the foundational contributions while focusing on the unique ethical, legal and practical challenges that arise at the intersection of innovation and patient care that support the safe, equitable and effective integration of AI into healthcare systems.

## 3. Key ethical principles in AI for healthcare

As AI continues to play a pivotal role in healthcare, it is essential to apply key ethical principles to ensure that these technologies are used in a manner that respects patients’ rights, promotes fairness, and minimizes harm. The integration of AI into healthcare raises important ethical concerns that must be addressed to safeguard patient well-being and maintain public trust. Below are the core ethical principles relevant to AI applications in healthcare.

### 3.1. Autonomy

Autonomy is a foundational principle in healthcare ethics, emphasizing a patient’s right to make informed decisions about their own care. In the context of AI, this principle becomes particularly important when AI systems are used to support or even make clinical decisions [45,46]. For instance, AI-driven diagnostic tools and treatment recommendations can influence medical decision-making, but patients must retain control over their healthcare choices.

One of the critical challenges in maintaining patient autonomy is ensuring informed consent in AI-supported processes [47,48]. Patients must be fully aware of how AI technologies are being used in their diagnosis or treatment and understand the implications of these tools on their care. This includes informing patients about the role AI plays in decision-making, any limitations or uncertainties associated with AI predictions, and their right to seek second opinions or opt for alternative treatment options. Clear communication about the function, accuracy, and limitations of AI systems is crucial to maintaining patient trust and ensuring that their choices remain informed and voluntary.

### 3.2. Transparency and explainability

AI systems, particularly those using deep learning, are often regarded as ‘black boxes’ due to their complexity and lack of transparency in decision-making processes [49]. However, the principle of transparency and the need for explainability are crucial to ensuring that AI is trusted by both patients and healthcare providers.

When AI systems are used in critical areas such as diagnostics or treatment planning, patients and healthcare providers need to understand how the AI arrived at its recommendations. This understanding fosters trust, allows healthcare professionals to make informed decisions about patient care, and ensures that patients feel confident in the technologies being applied to their treatment.

The explainability of AI also enables healthcare providers to assess the reasoning behind AI suggestions, empowering them to challenge or adjust decisions when necessary [50,51]. This is especially important in complex cases where clinical judgement and human experience should complement AI-generated insights. Research into interpretable AI and the development of tools that allow both clinicians and patients to understand the decision-making process of AI are vital to supporting ethical AI deployment in healthcare.

### 3.3. Accountability

As AI systems are increasingly integrated into clinical decision-making, establishing clear accountability for AI-driven decisions becomes essential [26,52]. In traditional healthcare settings, accountability for patient care decisions rests with the healthcare providers. However, when AI systems are involved in the decision-making process, it can be unclear who is responsible when things go wrong—whether it is the AI developers, the healthcare providers, or the institutions using the AI tools.

This is particularly complex when AI is used in hybrid human–AI decision-making processes, where both the AI system and the healthcare provider contribute to the final decision. Establishing clear guidelines for responsibility and liability is critical in ensuring that patients are protected and that the healthcare system remains accountable for the outcomes of AI-assisted care.

To address these concerns, policymakers and legal authorities must develop frameworks that define the roles and responsibilities of both AI developers and healthcare providers [28,53]. These frameworks should ensure that healthcare providers are adequately trained in the use of AI systems, that patients are informed about how decisions are being made, and that accountability is clearly established in cases of errors, malpractice, or adverse events.

## 4. Legal challenges in the deployment of AI in healthcare

As AI continues to reshape healthcare, the integration of AI systems presents a host of complex legal challenges that must be addressed to ensure both innovation and patient safety. The legal landscape surrounding AI in healthcare is multifaceted, involving issues related to data privacy and security, liability, regulatory approvals, intellectual property and cross-border regulations. Navigating these challenges is essential to the responsible deployment of AI technologies in healthcare settings.

### 4.1. Existing legal frameworks and their applicability to AI in healthcare

It is essential to recognize that many existing technology-agnostic laws and policies already provide a foundational framework for their governance. For instance, data privacy laws such as the GDPR in the EU [54] and the Health Insurance Portability and Accountability Act in the United States (US) [55] impose stringent requirements on handling sensitive patient data, irrespective of the technology used. These laws address issues like data protection, informed consent and the right to access and correct personal information, all of which are highly relevant to AI applications.

Furthermore, data are fundamental to AI development, especially in healthcare, where advanced AI models can revolutionize diagnostics, treatment and patient care. Creating these models requires substantial data and computational resources. Many companies lack the capacity to build their own AI models, relying instead on ‘model-as-a-service’ providers that develop and host models for third-party use via application programming interfaces. While such services enable businesses, including healthcare providers, to leverage AI, the continuous need for data to refine these models can conflict with privacy obligations. Users may inadvertently share sensitive or proprietary information, raising concerns about privacy breaches and misuse of competitive or patient data. In healthcare, this risk is heightened given the sensitivity of medical records and patient confidentiality. The US Federal Trade Commission [56] enforces strict compliance, holding model providers accountable for misusing customer data, including using it to train models without consent. Violations may result in mandates to delete unlawfully derived products. This underscores the importance of balancing AI innovation with robust data protection practices, particularly in health applications where trust and ethical considerations are paramount.

Human rights frameworks, including the Universal Declaration of Human Rights [57], emphasize principles such as equality, non-discrimination and the right to health. These principles are critical in addressing the potential of AI to amplify inequities or introduce bias into healthcare delivery. These existing frameworks, though not explicitly designed for AI, establish boundaries that can guide its development and deployment. However, the unique characteristics of AI, such as its opacity, scalability and potential for autonomous decision-making, necessitate adaptive extensions to these laws to ensure comprehensive governance.

### 4.2. Data privacy and security

One of the most significant legal concerns surrounding the deployment of AI in healthcare is data privacy [21,28,58]. AI systems require access to large volumes of sensitive patient data, including medical histories, genetic information and personal identifiers. As a result, AI developers and healthcare providers must ensure compliance with stringent data protection laws to safeguard patient confidentiality and privacy.

In the EU, the General Data Protection Regulation (GDPR) governs the processing of personal data, including health data, which is considered particularly sensitive. GDPR mandates that patient data is processed lawfully, transparently and for specific purposes, with patients having the right to access, correct, and delete their data. Furthermore, the regulation requires that any AI system involving personal data implements strong data security measures to prevent breaches and unauthorized access.

In the United States, the Health Insurance Portability and Accountability Act (HIPAA) sets forth similar requirements for protecting patient information, ensuring that healthcare providers and AI developers follow strict protocols for storing, sharing and accessing healthcare data. Failure to comply with GDPR, HIPAA, or other regional data protection laws can result in severe penalties, legal liabilities and loss of patient trust.

Given the sensitive nature of healthcare data, AI developers and healthcare providers must implement robust data security measures, including encryption, access controls and anonymization, to prevent unauthorized access and data breaches [58–60]. Additionally, AI systems must be designed to comply with these regulations from the outset, ensuring privacy is maintained throughout the AI lifecycle [23,61].

### 4.3. Liability and malpractice

Another significant legal challenge in AI-assisted healthcare is determining liability in the event of errors or adverse outcomes [40,62]. Traditional medical malpractice law holds healthcare providers accountable for patient care decisions [63,64]. However, when AI systems are involved in the decision-making process, it can be unclear who is responsible for errors or poor outcomes: the healthcare provider, the AI developers or the healthcare institution?

AI systems, particularly those based on machine learning, can learn from vast datasets and evolve over time. As a result, it becomes difficult to predict how the system might behave in every clinical situation. If an AI system provides an incorrect diagnosis or treatment recommendation that leads to patient harm, determining who is liable can become legally complex. In some cases, the AI may have been trained on biased or incomplete data [25,65], leading to suboptimal outcomes, while in others, the healthcare provider may have relied too heavily on the recommendations of AI without applying clinical judgement.

To address these challenges, legal frameworks must establish clear guidelines for shared responsibility in hybrid human–AI decision-making processes. This may involve attributing a portion of liability to the AI developers for flawed algorithms, while also holding healthcare providers accountable for their decisions in using AI systems. Additionally, healthcare institutions must ensure that AI systems undergo rigorous testing, validation and continuous monitoring to minimize errors and improve patient safety.

### 4.4. Regulatory approvals

The regulatory approval process for medical devices and technologies, including AI systems, is a crucial aspect of ensuring patient safety and efficacy. In many countries, AI technologies used in healthcare must be approved by regulatory bodies such as the US Food and Drug Administration (FDA) or the European Medicines Agency (EMA) before they can be deployed in clinical settings.

However, current challenges and gaps exist in certifying AI technologies due to the unique nature of these systems. Unlike traditional medical devices, AI-driven tools often evolve over time as they are trained on new data, making it difficult to establish a fixed, regulatory approval process [28,66,67]. This dynamic nature of AI systems presents a challenge for regulators, who must ensure that AI tools remain safe and effective throughout their lifecycle, not just at the time of initial certification.

Furthermore, the existing regulatory frameworks for medical devices were not designed with AI in mind [68,69], leading to gaps in how AI systems should be evaluated for approval. For example, the decision-making transparency and explainability of AI are crucial factors that may not be adequately addressed in current regulatory standards. Regulators must update existing frameworks to ensure that AI technologies meet the required standards for safety, efficacy and transparency, while also accounting for the dynamic, evolving nature of these systems.

China is a global leader in AI, especially in healthcare. The National Medical Products Administration (NMPA) has streamlined regulatory processes to accelerate AI-powered medical device approvals, aligning with China’s strategy to address public health challenges. Government guidelines focus on patient safety, ethical data use and AI performance monitoring [70]. However, the emphasis on rapid technological progress sometimes poses challenges in balancing innovation with patient rights [71].

India’s AI in healthcare is guided by the National Digital Health Blueprint (NDHB), which promotes ethical AI use and equitable care. The draft Personal Data Protection Bill emphasizes data sovereignty, requiring sensitive health data to be stored domestically. This aims to protect patient privacy and build trust in AI solutions. India also focuses on scalable, cost-effective AI innovations, like diagnostic tools for underserved rural areas, tailoring AI governance to address public health needs [72]. A key challenge is ensuring the effective implementation of AI solutions while addressing data privacy concerns and infrastructure limitations in rural areas [73].

Japan’s adaptive regulatory framework for AI in healthcare, overseen by the Pharmaceuticals and Medical Devices Agency (PMDA), emphasizes both innovation and patient safety in healthcare. The use of regulatory sandboxes allows for controlled testing of new technologies, fostering flexibility in AI development [74]. Japan’s focus on advanced healthcare integration, particularly in medical imaging and patient monitoring, provides valuable insights into balancing oversight with technological advancement. A challenge for Japan lies in ensuring that its regulatory framework can keep pace with the rapid evolution of AI technologies while maintaining patient safety and ethical standards [75].

### 4.5. Intellectual property

As AI becomes more integrated into healthcare, intellectual property concerns are becoming increasingly important. AI systems often rely on proprietary algorithms and data models that are developed by private companies or research institutions. The issue of ownership of these innovations raises complex ethical and legal questions, particularly in a field where the goal is to improve public health.

Who owns the intellectual property rights to AI systems that are used in healthcare? Is it the developers who created the algorithms, the healthcare providers who deploy the technology or the patients whose data were used to train the system? These questions become particularly contentious in cases where AI systems are developed with public funding or data sourced from diverse populations.

Moreover, there are ethical concerns regarding proprietary algorithms in healthcare [76–78]. The lack of transparency in proprietary AI systems may prevent healthcare providers from fully understanding how decisions are made, potentially undermining trust. Additionally, proprietary models can limit access to AI-driven innovations, as only organizations with the financial resources to license these technologies can benefit from their use. There is a need for open-source frameworks and collaborative innovations that ensure AI technologies are accessible, ethical and equitable.

### 4.6. Cross-border regulations

AI technologies are increasingly being used across borders, raising significant challenges in the form of cross-border regulations [79–81]. Different countries and regions have varying legal frameworks for the deployment of AI in healthcare, which can create inconsistencies and regulatory gaps. For instance, while the FDA and EMA have started to address AI in healthcare, these regulatory bodies have different standards for certification, approval processes and post-market surveillance.

This discrepancy in global regulatory standards can create challenges for multinational healthcare providers, AI developers, and patients. For example, a company that develops an AI-powered diagnostic tool may face difficulties navigating different regulatory requirements in the US, the EU and other regions, potentially delaying access to life-saving technologies.

To address these issues, there is a need for greater international cooperation in establishing global standards for AI in healthcare [34,82]. Collaborative efforts among regulatory bodies, healthcare providers and international organizations can help harmonize regulatory processes, ensuring that AI technologies meet safety, efficacy and ethical standards across borders. Moreover, creating universal guidelines for AI data privacy, transparency and accountability will help mitigate risks and ensure that AI innovations are deployed ethically and effectively worldwide.

## 5. Bias and fairness in healthcare AI

As AI becomes increasingly integrated into healthcare systems, ensuring fairness and addressing bias in these technologies is paramount. AI models are only as good as the data they are trained on, and if the training data is biased or unrepresentative, it can lead to unfair outcomes that disproportionately affect certain patient groups, particularly marginalized or underserved populations. The ethical and practical consequences of such biases are significant, as they can lead to misdiagnoses, unequal access to healthcare, and exacerbated health disparities. This section explores the sources of bias, strategies for mitigating it, and case studies that illustrate the real-world implications of bias in healthcare AI.

### 5.1. Sources of bias

Bias in healthcare AI primarily arises from the data that is used to train machine learning models. Several factors contribute to biased data, which can subsequently lead to discriminatory outcomes in patient care as follows.

#### 5.1.1. Historical bias

Many AI models are trained on historical healthcare data, which can contain ingrained biases reflecting past disparities in medical treatment [83]. For example, certain populations (e.g. racial minorities, women and low-income individuals) may have received inferior treatment or been under-represented in clinical studies. These historical biases are inadvertently reflected in the data, leading to AI models that perpetuate these inequalities.

#### 5.1.2. Data imbalance

AI models require large datasets to make accurate predictions, but healthcare data are often imbalanced. If certain demographic groups (such as minority racial or ethnic groups) are under-represented in training datasets, the AI model may fail to recognize conditions or make accurate predictions for these groups. For instance, a diagnostic tool trained predominantly on data from White patients may perform poorly when applied to patients of other races or ethnicities, resulting in misdiagnoses [84].

#### 5.1.3. Measurement bias

Bias can also emerge from how data is collected and measured. If certain conditions or symptoms are more likely to be recorded for certain populations (e.g. men versus women, White versus Black patients), AI systems may incorrectly associate those conditions with specific groups, leading to skewed predictions. For example, research has shown that algorithms used in determining healthcare prioritization may prioritize White patients over Black patients due to biased coding practices or differences in symptom presentation not adequately captured [39,85].

#### 5.1.4. Labelling bias

In supervised learning models, the labels provided to data (such as diagnoses or outcomes) can also introduce bias [86,87]. If labels are biased by human judgment or structural inequalities in the healthcare system, the AI system will learn and propagate those biases.

### 5.2. Mitigation strategies

To mitigate bias and ensure fairness in AI models, several strategies can be employed throughout the design, development and deployment stages as follows.

#### 5.2.1. Inclusive and diverse datasets

One of the most critical steps in mitigating bias is ensuring that the data used to train AI models is representative of all patient populations [25,88]. This means collecting data from diverse demographic groups, including different races, genders, ages, socioeconomic backgrounds and geographic locations. Inclusive datasets allow AI models to learn patterns and trends that are applicable across diverse patient populations, improving their accuracy and fairness in healthcare applications.

#### 5.2.2. Algorithm audits

Regular audits of AI models can help identify and address any potential biases in their predictions or decisions [89]. These audits typically involve analyzing how the AI performs across different demographic groups and assessing whether certain groups are systematically disadvantaged. Conducting these audits should be a standard practice before deploying AI systems in clinical settings, as it helps detect disparities and fine-tune the models for fairness.

#### 5.2.3. Fairness-aware design

AI developers can incorporate fairness into the very design of the algorithms [90]. This involves using techniques that explicitly account for fairness during the model training process. For instance, fairness constraints can be introduced to ensure that predictions do not disproportionately favor one group over another. Some methods focus on minimizing disparate impact, while others optimize for equal opportunity (i.e. ensuring that the model accuracy is similar for all groups). Additionally, different concepts of fairness and their associated metric can be integrated into the evaluation process to quantify fairness and guide adjustments [91].

#### 5.2.4. Transparency and explainability

Ensuring that AI models are transparent and interpretable is crucial for detecting and addressing bias [92]. When AI systems are understandable to healthcare providers and patients, it becomes easier to identify and correct instances of unfair decision-making. By fostering explainability, developers can ensure that healthcare professionals can review AI outputs, assess their fairness and make informed decisions based on both the predictions and clinical judgement of AI.

#### 5.2.5. Continuous monitoring and feedback loops

After deployment, continuous monitoring of AI systems is essential to ensure they remain fair over time [93]. Regular feedback loops, in which patient outcomes are assessed and compared across different demographic groups, can help identify any emerging biases and allow for ongoing adjustments to the AI model.

### 5.3. Case studies

Several high-profile cases have highlighted how bias in AI can lead to harmful healthcare disparities. These case studies illustrate the need for vigilance and the implementation of fairness strategies in AI design.

#### 5.3.1. COMPASS recidivism risk algorithm

The COMPASS algorithm [94], used in the US to predict the likelihood of reoffending in criminal justice, was found to disproportionately flag Black defendants as high-risk compared to White defendants, despite similar crime histories. Although this algorithm is not directly related to healthcare, the case is relevant because it highlights how predictive models can perpetuate systemic racial biases. Similar biases could occur in healthcare AI if models are trained on unrepresentative data or on data that reflects societal inequities. For example, a risk-assessment tool used in healthcare to predict a patient’s likelihood of developing a condition could overestimate risk for minority groups, leading to overtreatment or misdiagnosis.

#### 5.3.2. Optum’s healthcare risk prediction algorithm

A study published in *Science* [39] in 2019 uncovered that Optum’s AI-driven healthcare algorithm, which was designed to identify high-risk patients for healthcare intervention, systematically disadvantaged Black patients. The algorithm was trained on healthcare spending rather than healthcare needs, which resulted in biases because Black patients often receive lower levels of care, leading to lower healthcare expenditures. As a result, the system underestimated the health risks of Black patients, contributing to inequities in access to care and treatment. Following this revelation, Optum made changes to its algorithm to improve fairness and ensure that it more accurately identified at-risk patients, regardless of their race.

#### 5.3.3. IBM Watson for oncology

IBM Watson’s AI tool designed to assist oncologists in diagnosing and recommending treatments for cancer was found to provide unsafe and ineffective treatment recommendations in some cases [95]. Although not explicitly a bias issue related to demographic groups, the failure of Watson was partly attributed to biased training data. Watson had been trained on data from a limited number of hospitals, which may have led it to make inappropriate recommendations in certain clinical contexts. The case highlighted the importance of diverse and representative datasets in training AI models for healthcare.

#### 5.3.4. Facial recognition algorithms in healthcare

A study published in 2018 [96] demonstrated that commercial facial recognition software, including AI systems used in healthcare, was less accurate at identifying the faces of Black and Asian subjects compared to White subjects. This raised concerns about the potential for such algorithms to be used in settings such as patient identification, where biased recognition could lead to errors in treatment or mistreatment of marginalized populations. These findings underscore the critical need for fairness-aware design and the need to consider potential biases in AI systems, particularly when they directly impact patient safety and access to care.

## 6. AI in personalized medicine: ethical and legal issues

Personalized medicine, driven by advancements in AI, promises to revolutionize healthcare by tailoring treatment plans to individual patients based on their genetic, environmental and lifestyle factors [9,97,98]. The ability of AI to analyse vast amounts of patient data to predict optimal treatments is unparalleled, but this transformation also brings significant ethical and legal tensions. The use of AI in personalized medicine raises critical questions surrounding patient privacy, the ethical and legal handling of genetic data, and the challenge of ensuring equitable access to these innovations. This section explores these issues and how they can be addressed to ensure the responsible application of AI in personalized medicine.

### 6.1. Balancing patient privacy with the need for personalized data in AI-driven treatment

One of the foundational principles of healthcare is patient privacy—the right of individuals to have control over their personal health information [99]. However, in personalized medicine, AI requires access to a vast array of sensitive data, including genetic information, biological markers, lifestyle factors and clinical histories, all of which are essential for developing personalized treatment plans. Balancing privacy concerns with the need for this data is a delicate ethical and legal issue.

#### 6.1.1. Informed consent

Central to maintaining privacy is the concept of informed consent [100]. Patients must be fully informed about the types of data being collected, how it will be used, and the potential risks to their privacy. In the context of AI-driven personalized medicine, informed consent should extend beyond the standard procedural information, encompassing how AI algorithms will analyse the data, how decisions will be made, and whether those decisions could have an impact on their treatment or access to care. Ensuring that patients understand the potential scope and reach of AI systems in personalized medicine is crucial.

#### 6.1.2. Data anonymization

To protect patient privacy, anonymization of personal health data can be a key approach. However, even anonymized data can sometimes be re-identified [101], especially with the increasing sophistication of AI algorithms. This raises concerns about the limits of anonymization and the security measures required to ensure that patient identities are protected. Ethical questions emerge when patients’ anonymized data is used without their explicit consent for secondary purposes, such as research or commercial use, which may not align with their original consent.

#### 6.1.3. Data sharing and security

Personalized medicine often involves collaboration between multiple stakeholders, such as healthcare providers, researchers and pharmaceutical companies. In this context, ensuring that data sharing occurs securely and ethically is critical. AI systems that aggregate and analyse large datasets from different sources must comply with data protection regulations to prevent unauthorized access and misuse. Legal frameworks must evolve to address the complexities of data ownership, sharing and consent in the realm of AI-driven personalized healthcare.

### 6.2. Legal and ethical frameworks governing the use of genetic data in precision medicine

Genetic data play a pivotal role in precision medicine, offering insights into a patient’s susceptibility to diseases, likely treatment responses and potential drug reactions. However, the collection, storage and use of genetic data bring forth significant ethical and legal challenges that require robust frameworks.

#### 6.2.1. Genetic data and ownership

One of the most debated issues in personalized medicine is the ownership of genetic data [102]. Who owns an individual’s genetic information? Is it the patient, the healthcare provider or the company that sequences and analyses the data? Legal ambiguity around the ownership of genetic data can complicate matters such as data access, data sharing and informed consent. For instance, genetic data collected for research purposes may be used in AI models without the patient’s explicit consent for all future uses, raising questions about data ownership and control.

#### 6.2.2. Non-discrimination

The use of genetic information in personalized medicine must also adhere to ethical principles of non-discrimination [103]. Legal protections, such as the Genetic Information Nondiscrimination Act in the US [104], prohibit the use of genetic data for discrimination in employment and insurance. However, these protections are not universal across all countries, and there are gaps in legal frameworks regarding the protection of individuals from discrimination based on genetic information in other areas of life, such as education or healthcare. Ethical concerns also arise about the potential for genetic data to be used to exclude patients from certain treatments or healthcare coverage based on their genetic predispositions.

#### 6.2.3. Genetic data for research

AI-driven precision medicine often relies on large datasets, including genetic data, to build predictive models and inform treatment decisions [9,105]. However, using genetic data for research purposes raises significant ethical concerns about consent and privacy. Patients may consent to their genetic data being used for one purpose, but the use of that data for secondary purposes, such as research or development of commercial products, may require additional consent. Moreover, ensuring the confidentiality of genetic data is paramount, as the data are inherently unique and can be re-identified, potentially exposing patients to genetic risks or discrimination.

### 6.3. The challenge of ensuring equitable access to AI-driven personalized healthcare solutions

While AI promises to improve the precision and efficacy of treatments, there are ethical concerns about ensuring that these benefits are accessible to all patients, particularly those from underserved or marginalized communities. Personalized medicine powered by AI has the potential to worsen health disparities if equitable access is not ensured.

#### 6.3.1. Socioeconomic disparities

Access to cutting-edge AI-driven personalized treatments may be limited by socioeconomic factors. Patients in low-income or rural areas may lack access to the latest technologies, genetic testing or AI-driven treatment options, which are often concentrated in well-funded urban healthcare centres or private institutions. This disparity can lead to unequal health outcomes, where wealthier patients benefit from advanced treatments while others are left with suboptimal care.

#### 6.3.2. Healthcare infrastructure

Implementing AI-driven personalized medicine requires significant healthcare infrastructure—including AI technologies, trained professionals and data systems—most of which are concentrated in developed countries or affluent areas. This unequal distribution of resources can limit access to personalized healthcare solutions for populations in developing nations or lower-income regions. Governments and healthcare organizations need to work together to create strategies that ensure the benefits of AI in healthcare are widely distributed.

#### 6.3.3. Bias in AI models

As discussed in earlier sections, AI models are at risk of reflecting and perpetuating biases in the data they are trained on. If these models are trained on datasets that predominantly represent certain demographics (e.g. middle-aged, White, Western patients), they may not perform as effectively for other patient groups. This introduces an equity gap in healthcare, where AI-driven treatments may be less effective for minority groups, exacerbating health inequities.

#### 6.3.4. Regulatory challenges

Different countries have varying regulations concerning the use of AI in personalized medicine [82,106], leading to disparities in access based on geographical location. For example, AI technologies that are FDA-approved in the US may not be approved or available in other countries. In addition, the cost of developing and deploying AI technologies in personalized medicine can be prohibitively high, creating barriers to entry for healthcare systems in lower-income countries.

To address these challenges, it is crucial to implement policies and frameworks that prioritize equitable access to AI-driven personalized healthcare. This includes efforts to reduce cost barriers, improve healthcare infrastructure, and ensure that AI models are developed with diverse and representative datasets to ensure they are effective across all demographic groups.

## 7. Policy implications

As AI technologies become increasingly integral to healthcare delivery, policymakers are faced with the challenge of developing and implementing ethical and legal frameworks that ensure the responsible, equitable and safe deployment of AI in healthcare systems [28,40]. Developing such frameworks requires careful consideration of the potential benefits, risks and complexities of AI, while ensuring that the needs of patients, healthcare providers and society at large are met. This section explores key policy proposals, the role of public–private partnerships, the importance of international cooperation and the need for adaptive regulation in shaping a robust AI healthcare policy.

### 7.1. Frameworks

To guide the ethical and legal deployment of AI in healthcare, policymakers need to establish clear and comprehensive frameworks. These frameworks should encompass several key elements as follows.

#### 7.1.1. Global health standards

Given the transformative potential of AI across borders, it is essential to establish global health standards that ensure consistency in the use of AI in healthcare [107]. These standards should address issues such as patient privacy, data protection, clinical validation, and safety protocols for AI systems. Standards like those developed by the World Health Organization (WHO) and International Telecommunication Union (ITU) can offer guidelines for the integration of AI in healthcare systems worldwide. These global standards must be flexible enough to accommodate the diverse healthcare systems in different countries, while maintaining a core commitment to patient-centred care.

#### 7.1.2. Interoperability

AI systems in healthcare must be able to interoperate seamlessly with existing healthcare technologies and databases [1,108]. Policymakers should push for standards that ensure data compatibility between AI systems, electronic health records (EHRs), and other medical technologies. This will facilitate the exchange of patient information, enhance decision-making and promote better coordination of care. Additionally, regulations should address the need for data standardization, ensuring that AI systems are able to work across various platforms, healthcare providers and geographical boundaries.

#### 7.1.3. Transparency mandates

Transparency is critical to fostering trust in AI technologies. Policymakers should mandate transparency in how AI systems operate and how decisions are made, especially in patient care. This includes requiring clear documentation of the training data used to build AI models, the algorithmic decision-making processes, and outcome predictions. Transparent AI systems allow healthcare professionals to understand the reasoning behind AI-driven decisions, which is crucial for clinical validation, especially in high-stakes environments like healthcare.

#### 7.1.4. Ethical design and impact assessment

Policymakers should encourage the integration of ethical considerations in the design and implementation of AI systems. This includes ensuring that AI models are bias-free, fair and equitable, and that they promote patient autonomy and non-maleficence. Ethical guidelines should also require impact assessments for AI technologies before they are deployed, ensuring that their potential impact on patient outcomes and healthcare systems is thoroughly evaluated.

### 7.2. Public–private partnerships

The rapid advancement of AI in healthcare necessitates collaboration between governments, regulatory bodies and the private sector to ensure that ethical and legal standards are developed, implemented, and enforced [28]. Public–private partnerships can play a critical role in the responsible deployment of AI in healthcare by:

#### 7.2.1. Driving innovation with ethical boundaries

Collaboration between governments and private companies can lead to the development of innovative AI solutions while maintaining ethical boundaries. For example, healthcare startups may provide the cutting-edge technology needed for AI applications, while governments can ensure that those technologies align with ethical guidelines and regulatory requirements. This collaboration can help bridge the gap between the technical capabilities of AI and the ethical concerns inherent in its use.

#### 7.2.2. Setting industry standards

Governments can work with private companies, research institutions and professional organizations to develop industry standards for AI in healthcare. This includes establishing guidelines for the ethical collection and use of patient data, ensuring fair access to AI technologies and promoting equity in the development and application of AI systems. By involving stakeholders across the public and private sectors, policymakers can ensure that industry standards reflect the diverse interests and concerns of healthcare providers, patients and AI developers.

#### 7.2.3. Regulation and enforcement

Governments can also play a critical role in regulating and enforcing ethical standards for AI technologies. Legislative bodies can create clear policies that require companies to adhere to ethical principles in their AI development and deployment processes, with enforceable penalties for non-compliance. Industry stakeholders, in turn, can assist in ensuring that regulations are practical, feasible and conducive to innovation.

### 7.3. International cooperation

Given the borderless nature of AI technologies, international cooperation is critical to ensure the ethical, legal, and equitable deployment of AI in healthcare worldwide [53]. As AI-driven healthcare solutions become more prevalent, cross-border collaboration can help ensure that AI is used responsibly and consistently across different healthcare systems.

#### 7.3.1. Unified regulatory frameworks

Policymakers should work together across national borders to develop unified regulatory frameworks for AI in healthcare. This could involve the creation of international regulatory bodies or collaborations between existing organizations such as the WHO and the International Organization for Standardization (ISO). By aligning national regulations and fostering mutual recognition of standards, governments can reduce barriers to the adoption and diffusion of AI technologies across countries and ensure that AI-driven healthcare solutions meet consistent ethical and legal criteria.

#### 7.3.2. Cross-border data sharing and security

AI technologies in healthcare often rely on large datasets, which may be sourced from multiple countries [2,109]. Establishing global data-sharing agreements will be essential to enable the development of effective AI models while ensuring that data protection regulations are upheld. International treaties or agreements could govern the secure sharing of patient data, ensuring that patient privacy is respected, and that data is used ethically across jurisdictions.

#### 7.3.3. Equitable access to AI technologies

Global cooperation is also necessary to address the digital divide and ensure that AI-driven healthcare benefits all populations, regardless of geographical location. Wealthier nations should collaborate with low- and middle-income countries to provide resources, training and technology transfer that enable equitable access to AI-driven personalized healthcare. This could involve international funding initiatives or partnerships between governments, non-governmental organizations (NGOs) and the private sector to enhance healthcare access in underserved areas.

### 7.4. Adaptive regulation

AI technologies evolve rapidly, and regulatory frameworks must be agile enough to keep pace with these changes. Adaptive regulation involves developing iterative, flexible and future-proof policies that can be updated as AI technologies mature and new ethical and legal challenges arise.

#### 7.4.1. Responsive regulatory models

Rather than relying on rigid, one-time policies, regulators should adopt responsive frameworks that evolve alongside AI technologies. This could involve setting periodic reviews of AI healthcare applications, ensuring that regulations remain relevant and address emerging risks or opportunities. For example, real-time monitoring of AI systems could be implemented to ensure that they function as expected and do not cause unintended harm or bias over time [110].

#### 7.4.2. Collaborative regulatory bodies

Policymakers should establish regulatory bodies that involve AI experts, healthcare professionals, ethicists and patient advocacy groups. This collaborative approach will allow for a broader range of perspectives when developing regulatory policies and can help ensure that regulations reflect both the technical realities of AI and the ethical principles that guide healthcare delivery.

#### 7.4.3. Flexibility in AI validation and certification

Regulators should allow for flexible pathways in the validation and certification of AI technologies in healthcare [111,112]. For instance, initial approval could focus on specific AI applications (e.g. medical imaging or diagnostics), with ongoing validation as new functionalities or updates are introduced. This allows regulators to keep up with rapid technological developments while ensuring that patient safety and ethical concerns remain central.

## 8. Public trust and engagement

As AI becomes an integral part of healthcare systems [1,2,108], fostering public trust is paramount to its successful adoption and integration. Trust is essential for ensuring that patients, healthcare providers and the general public feel confident in the safety, efficacy and fairness of AI applications in healthcare settings. Public engagement plays a crucial role in building this trust, addressing concerns, and ensuring that AI technologies are developed and deployed in ways that align with the values and expectations of society. This section explores the role of public engagement in fostering trust, the importance of addressing ethical concerns, and reviews case studies of public–private initiatives aimed at building trust in AI innovations.

### 8.1. The role of public engagement in fostering trust in AI applications

Public engagement is a critical component of trust-building in AI, especially when it comes to technologies that directly impact individuals’ health and well-being. Engaging the public in discussions about the potential benefits and risks of AI allows for transparency, accountability, and inclusive decision-making. Public engagement strategies should include the following.

#### 8.1.1. Educational initiatives

Raising awareness and understanding of the potential of AI in healthcare through public education campaigns is essential for dispelling myths and misinformation [113]. By providing accurate and accessible information, healthcare organizations and governments can equip the public with the knowledge needed to make informed decisions about AI. This includes explaining how AI technologies work, their limitations, and how they benefit patient outcomes.

#### 8.1.2. Open dialogue

Facilitating open, two-way communication between AI developers, healthcare professionals, and the public fosters trust by giving people a platform to voice their concerns and questions. Public consultations, town hall meetings and surveys can help policymakers and AI developers understand public perceptions, fears and expectations regarding AI in healthcare.

#### 8.1.3. Community involvement in decision-making

Incorporating diverse public stakeholders in decision-making processes about AI policies and applications ensures that a wide range of perspectives is considered [114]. This can include patient advocacy groups, ethical committees, and marginalized communities. When people feel involved in decisions that affect their healthcare, they are more likely to trust AI technologies and the institutions that deploy them.

### 8.2. Importance of addressing public concerns about AI ethics, privacy and decision-making power in healthcare

For AI to gain public trust, ethical concerns must be addressed comprehensively. Several key areas of concern are as follows.

#### 8.2.1. Ethical considerations

The public often worries about whether AI will make decisions that align with their values, especially in healthcare, where personal well-being is at stake [115,116]. Questions about bias in AI algorithms, the autonomy of decision-making, and the potential for discrimination can lead to reluctance in trusting AI systems. Addressing these concerns requires ensuring that AI systems are developed with ethical frameworks that prioritize non-maleficence (doing no harm), beneficence (maximizing benefit) and justice (ensuring fairness).

#### 8.2.2. Privacy and data protection

Patients are increasingly wary about how their personal health data will be used, stored and shared [117]. Ensuring compliance with regulations such as GDPR in Europe or HIPAA in the US, and offering robust data protection mechanisms, can help mitigate fears. Transparent communication about how data is handled, what it is used for and how it is kept secure is essential for addressing privacy concerns.

#### 8.2.3. Decision-making power

Many people are concerned about AI systems taking over decision-making in healthcare, fearing that machines may replace the judgement of healthcare professionals [118]. Clear communication about the human–AI collaboration model, where AI assists but does not replace healthcare providers, can help assuage these fears. Ensuring that AI is viewed as a tool that empowers professionals rather than replacing them is key to fostering trust.

#### 8.2.4. Accountability for errors

When AI is involved in healthcare decisions, questions about accountability become crucial [26,119]. Who is responsible if something goes wrong? Is it the healthcare provider, the AI developer or the institution using the AI system? Ensuring clear lines of accountability and addressing concerns about liability and malpractice in the context of AI-driven healthcare can help build trust in the system.

### 8.3. Case studies of public–private initiatives aimed at building trust in AI innovations

Several public–private initiatives have successfully engaged the public and addressed concerns about AI in healthcare. These case studies demonstrate how collaboration between government bodies, private companies and healthcare organizations can build trust and ensure that AI technologies are adopted responsibly.

#### 8.3.1. The UK’s national AI strategy

The UK government has launched initiatives under its National AI Strategy, which focuses on engaging the public in discussions about AI future in healthcare and other sectors [120]. This strategy includes public consultations, ethical guidelines for AI development and data governance frameworks that address privacy concerns. By actively involving the public in shaping AI policies and regulations, the government aims to foster trust in AI technologies while ensuring they are developed in ways that prioritize fairness, transparency and patient safety.

#### 8.3.2. AI in healthcare

Public and Private Collaboration (US): an excellent example is the public–private partnership between the FDA and private tech companies to develop frameworks for AI regulatory approval in healthcare [121]. The initiative includes pilot programs designed to test AI tools in real-world clinical settings while engaging patients and healthcare providers to gather feedback on the effectiveness and ethical considerations of AI. This initiative helps ensure that AI tools are developed with patient safety in mind while also building public confidence in AI-driven innovations.

#### 8.3.3. AI for good initiative (United Nations)

The AI for Good initiative [122], led by the United Nations, promotes the use of AI to address global health challenges, including healthcare access and disease prevention. By engaging governments, NGOs, the private sector and local communities, this initiative fosters trust in AI’s potential to improve healthcare outcomes in under-served regions. It also emphasizes the importance of ethical considerations in AI deployment, ensuring that AI technologies are used for the benefit of all populations, not just the privileged.

## 9. Future directions

As AI continues to evolve, its integration into healthcare is expected to deepen, offering new solutions and possibilities. However, with the rapid pace of technological advancement, significant ethical and legal challenges will emerge. These challenges will require adaptive policies, continuous ethical evaluation and robust legal frameworks to ensure that the potential of AI is harnessed in ways that prioritize patient safety, equity and transparency.

Table 1 highlights common ethical challenges associated with AI applications in healthcare, providing examples of specific technologies and their implications. Table 2 presents an overview of regulatory approaches to AI in healthcare across various countries, illustrating how different jurisdictions address these emerging ethical and legal considerations.

**Table 1.** Examples of AI applications in healthcare and their ethical implications.

AI application	description	ethical considerations
diagnostics	AI algorithms that aid in early detection of diseases such as cancer	ensuring accuracy, mitigating biases, and providing transparent results to patients and providers
personalized medicine	tailoring treatments based on individual genetic profiles and health data	balancing patient privacy with data sharing for accurate recommendations, ensuring equitable access to AI tools
robotic surgery	AI-driven surgical tools enhancing precision and reducing recovery times	accountability for surgical outcomes, informed consent for AI-assisted procedures
medical imaging	AI for enhanced image analysis in radiology, pathology and other areas	transparency in AI decision-making, potential over-reliance on AI by healthcare professionals
drug discovery	accelerating drug discovery by predicting molecule interactions and outcomes	ownership of innovations, ensuring public access to AI-enabled treatments
telemedicine	virtual healthcare consultations enhanced by AI for diagnosis and treatment	data privacy in virtual consultations, fairness in access to digital healthcare, informed consent

**Table 2.** Regulatory approaches to AI in healthcare across different jurisdictions.

region	key regulatory body	regulatory focus	challenges in AI healthcare
United States	FDA (Food and Drug Administration) [123]	focus on the approval of medical devices and AI-driven diagnostics	current regulations not fully adapted to continuous learning AI systems
European Union	EMA (European Medicines Agency) [124]	stringent data privacy rules under GDPR, AI as part of medical device regulations	GDPR complexities affecting cross-border AI data sharing and patient privacy
United Kingdom	MHRA (Medicines and Healthcare Products Regulatory Agency) [125]	focus on software as a medical device (SaMD) and AI safety standards	navigating post-brexit regulatory environment and aligning with EU and global standards
China	NMPA (National Medical Products Administration) [126]	accelerated AI innovation in healthcare under government directives	balancing rapid AI adoption with patient safety, ensuring alignment with international laws
Japan	PMDA (Pharmaceuticals and Medical Devices Agency) [127]	focus on innovation-friendly regulations with an emphasis on public safety in healthcare	ensuring AI transparency and fairness while supporting technological innovation
global initiatives	WHO (World Health Organization) [128]	global guidance on ethical use of AI in healthcare, promoting safety, efficacy, and equity	lack of harmonized international regulations, particularly for cross-border AI technologies

The remainder of this section explores long-term policy implications, and the ethical and legal challenges that may arise as AI systems become more autonomous in clinical decision making and patient care.

### 9.1. Long-term implications for policy

As AI technology in healthcare accelerates, policymakers will face increasing challenges in ensuring that regulations keep pace with developments. Key policy considerations will include the following.

#### 9.1.1. Agile regulation

Given the fast-evolving nature of AI technologies, regulatory frameworks must be adaptable to new applications and innovations. Policymakers should consider implementing adaptive regulation, which can evolve with the technology. This approach would involve periodic reviews and updates to existing laws and guidelines to reflect the current state of AI and its implications. Ensuring that regulatory bodies can respond quickly to emerging technologies is crucial for preventing gaps in coverage or oversight.

#### 9.1.2. Equitable access to AI technologies

As AI becomes more central to healthcare, ensuring equitable access to these innovations will be critical. Policymakers must address issues of affordability, particularly in low-resource settings, where AI-driven healthcare solutions may be inaccessible. Additionally, there will be a need to develop policies that ensure marginalized communities, including racial and ethnic minorities, have equal access to AI-driven healthcare, avoiding the risk of deepening health inequities.

#### 9.1.3. Cross-border policy alignment

The global reach of AI means that healthcare systems across different countries must develop aligned standards to regulate AI in healthcare. Cross-border regulatory cooperation will be crucial in ensuring the safety and efficacy of AI technologies, particularly when they are deployed internationally or across multinational organizations. Policies must address the complexities of cross-border data exchange, data privacy laws and differing regulatory requirements.

### 9.2. Ethical and legal challenges with increasing AI autonomy in clinical decision-making

The autonomous or increasingly autonomous nature of AI systems in healthcare introduces unique ethical and legal complexities that extend beyond those associated with traditional, non-autonomous technologies. Unlike conventional systems, autonomous AI systems are capable of making diagnostic and treatment decisions with minimal human intervention, which significantly changes the dynamics of accountability, transparency, and informed consent. For example, traditional technologies often function as tools directly controlled by healthcare professionals, making it easier to trace responsibility for errors or adverse outcomes. In contrast, autonomous systems can operate independently, raising questions about who should be held accountable when these systems fail or make incorrect decisions.

Furthermore, the autonomous feature directly impacts patient consent and trust. Patients must not only understand the data being collected and used but also the extent of AI system autonomy in their care. Additionally, the lack of human oversight in certain autonomous systems may challenge existing legal frameworks, which typically assume a human decision maker.

Lastly, the self-learning capabilities of autonomous AI systems introduce an evolving risk landscape. These systems may change their behaviour over time based on new data, potentially leading to unforeseen outcomes that were not accounted for during initial deployment or approval. This necessitates continuous monitoring, robust validation mechanisms, and adaptive regulatory frameworks to address these evolving risks effectively.

Two key issues of ethical and legal challenges, which are likely to emerge, are outlined below.

#### 9.2.1. The challenge of informed consent

The challenge of informed consent in AI-driven healthcare encompasses both ethical and legal dimensions, underscoring the need for updated frameworks to address the complexities introduced by these technologies. Ethically, a key challenge arises: how can patients provide truly informed consent if they do not fully understand how the AI system operates, its limitations, or its decision-making processes? The lack of clarity and technical complexity of AI systems often hinder patients’ ability to make truly informed decisions, raising concerns about autonomy and trust. Legally, traditional consent frameworks may fall short in accommodating the dynamic nature of AI systems, particularly when they adapt over time or use patient data for continuous learning. This creates potential liabilities if patients are unaware of how their data is being used or cannot fully comprehend the implications of AI-driven decisions. Bridging these gaps requires transparent communication, ongoing education and regulatory updates that prioritize both ethical patient engagement and robust legal protections.

#### 9.2.2. The use of AI in end-of-life care

As AI begins to play a larger role in palliative care and end-of-life decision-making, significant ethical and legal concerns may arise [129–131]. Ethically, AI systems tasked with recommending life-sustaining treatments or end-of-life care plans based on clinical data must navigate deeply personal and subjective considerations that algorithms may struggle to address. Ensuring that such systems respect patient values, preferences, and autonomy is essential, and human clinicians will remain critical in making these delicate decisions. Legally, the use of AI in these contexts raises questions about liability, particularly if an AI recommendation leads to outcomes contrary to patient wishes or if it overlooks subtle clinical factors. Additionally, issues of informed consent become even more complex in end-of-life care, as patients or their families may need to understand the role of AI in shaping care plans. These challenges call for robust ethical guidelines and legal frameworks to ensure that AI supports rather than undermines compassionate and patient-centred care.

## 10. Conclusion

The integration of AI into healthcare holds transformative potential, but it also presents a range of ethical and legal challenges that must be carefully addressed. Key concerns include autonomy, accountability, data privacy, bias, informed consent and liability, all of which require robust frameworks to ensure AI technologies are used responsibly. The rapid development of AI necessitates multi-disciplinary collaboration among technologists, healthcare providers, legal experts and policymakers. This collective effort is essential for creating systems that prioritize patient safety, fairness and equity, while fostering innovation in a regulated and transparent environment. Aligning AI innovations with ethical principles and legal imperatives will be crucial for building public trust and ensuring that the benefits of AI are accessible to all, ultimately contributing to a more equitable and effective healthcare system.

## Data Availability

This article has no additional data.
